# Application of click chemistry in nanoparticle modification and its targeted delivery

**DOI:** 10.1186/s40824-018-0123-0

**Published:** 2018-04-13

**Authors:** Gawon Yi, Jihwan Son, Jihye Yoo, Changhee Park, Heebeom Koo

**Affiliations:** 1 Department of Medical Lifescience, College of Medicine, The Catholic University of Korea, 222 Banpo-daero, Seocho-gu, Seoul, 06591 Republic of Korea; 2 Catholic Photomedicine Research Institute, College of Medicine, The Catholic University of Korea, 222 Banpo-daero, Seocho-gu, Seoul, 06591 Republic of Korea

**Keywords:** Nanoparticle, Click chemistry, Bioorthogonality, Drug delivery

## Abstract

**Background:**

Click chemistry is termed as a group of chemical reactions with favorable reaction rate and orthogonality. Recently, click chemistry is paving the way for novel innovations in biomedical science, and nanoparticle research is a representative example where click chemistry showed its promising potential. Challenging trials with nanoparticles has been reported based on click chemistry including copper-catalyzed cycloaddition, strain-promoted azide-alkyne cycloaddition, and inverse-demand Diels-Alder reaction.

**Main body:**

Herein, we provide an update on recent application of click chemistry in nanoparticle research, particularly nanoparticle modification and its targeted delivery. In nanoparticle modification, click chemistry has been generally used to modify biological ligands after synthesizing nanoparticles without changing the function of nanoparticles. Also, click chemistry in vivo can enhance targeting ability of nanoparticles to disease site.

**Conclusion:**

These applications in nanoparticle research were hard or impossible in case of traditional chemical reactions and demonstrating the great utility of click chemistry.

## Background

Convergence is one of the favorite words in recent research field. Recently, challenging combination of multiple techniques from different research area has been widely tried to overcome the current hurdles. Biomedical application of nanoparticles, so called nanomedicine, is the representative result of the combinational researches based on chemistry, biology, material science, pharmaceutics, and clinic [1]. Organic materials such as polymers or phospholipids can self-assemble into spherical nano-structure in aqueous solution resulting micelles or liposomes [2, 3]. Inorganic materials including gold, iron oxide, or silica can provide controlled growth at nanoscale, and more complicated structures like rod, star, sheet, tube, or porous sphere can be obtained [4]. The advances in chemistry, material science, and nanotechnology enabled production of various kinds of interesting and well-defined nanoparticles which were attractive for biologists and biomedical scientists [5]. Until now, a huge number of nanoparticles have been developed as imaging probes or drug carriers, and some of them are used in clinic or pending in clinical trials [6].

Nanoparticles have shown several advantages as drug carriers. First, due to their large surface area or internal volume, large amount of drug can be loaded or conjugate into single nanoparticle. Second, multiple different molecules can be introduced into nanoparticles resulting in their multi-functionality [7]. Third, their size with several tens or hundreds nanometers can enable longer circulation time in body and slow the secretion of drug for better therapeutic outcome [8]. These advantages have made nanoparticles as promising and innovative drug carriers for the clinical fields. Particularly, in case of intravenous injection, nanoparticles provide high accumulation in angiogenic disease tissues including tumors based on the so-called enhanced permeation and retention effect (EPR) in fenestrate structures of newly generated blood vessels [9]. Furthermore, attachment of targeting ligands such as antibody, peptide, or aptamer can increase the binding of nanoparticles to the specific receptors on the surface of the target disease cells resulting in further enhance the targeting efficacy [10].

**Scheme 1:**
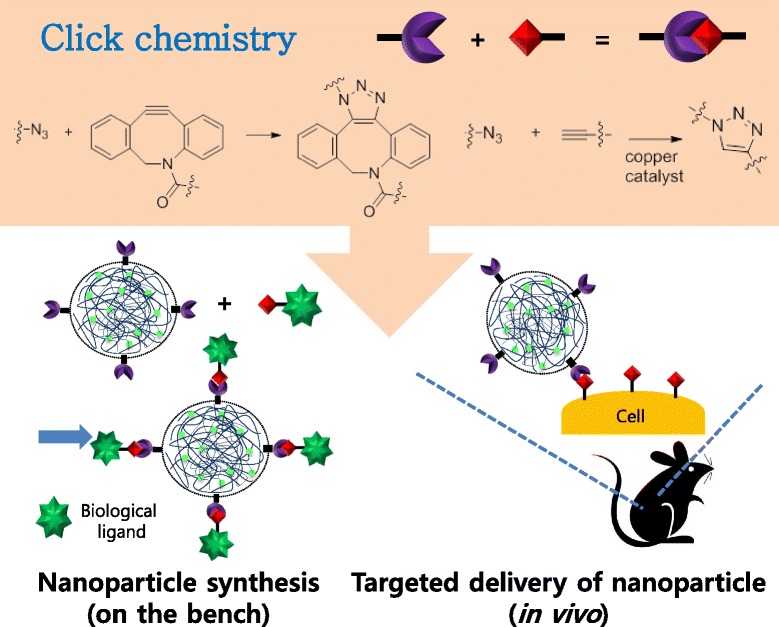
Illustration for the usage of click chemistry during nanoparticle synthesis and its targeting in vivo

Recently, a newly emerging technique, click chemistry has garnered a great deal of attention from researchers in biomedical science as well as in chemistry and material science [11]. Click chemistry is termed as a group of chemical reactions that have special advantages compared to traditional reactions [12]. It is orthogonal with other functional groups including amines, carboxylic acids, thiols, and so on. It has favorable reaction rate in aqueous condition, and generates minimal byproducts. Due to these advantages, click chemistry has been widely applied in various studies, and biomedical scientists also interested in its biocompatibility and bioorthogonality [13]. Copper-catalyzed cycloaddition between azide and alkyne is the first reaction that was called as click chemistry. After that, cycloaddition between strain-promoted alkyne and azide enabled copper-free click chemistry [14]. Furthermore, inverse-demand Diels-Alder reaction with tetrazine (Tz) and trans-cyclooctene (TCO) provided ungraded reaction rate [15]. These click chemistry has been a useful tool for nanoparticle research [16]. In this review, we will introduce the application of click chemistry in modification or targeted delivery of nanoparticles for imaging or drug delivery (Scheme 1).

### Application of click chemistry in nanoparticle research

#### Application in nanoparticle modification

During development of nanoparticles, click chemistry generally used to attach biological ligands to the surface of nanoparticles. Antibody is one of the most favorite ligands that used for a long time to increase binding to specific cell line. Particularly, antibody is a protein with large molecular weight, it is important not to affect its binding site. In 2012, Colombo et al. used strain-promoted azide-nitrone cycloaddition to attach their antibody to nanoparticle. They used scFv variant of the anti-HER2 antibody and introduced a serine at N-terminus of the scFv by genetic engineering [17]. The serine was oxidized into aldehyde by sodium periodate, and further changed into nitrone with help of *p*-methoxybenzenethiol, *p*-anisidine, and *N*-methylhydroxylamine. Finally, scFv was attached to dibenzocyclooctene-modified hybrid multifunctional nanoparticles by click chemistry with the resulting nitrone group. After complete process, the nanoparticles were dispersed well in PBS with slightly increased size due to protein conjugation. The scFv-conjugated nanoparticles showed enhanced binding to HER2-positive MCF7 cells compared to HER2-negative MDA cells demonstrating that the binding of antibody was not change after modification by click chemistry.

**Fig. 1:**
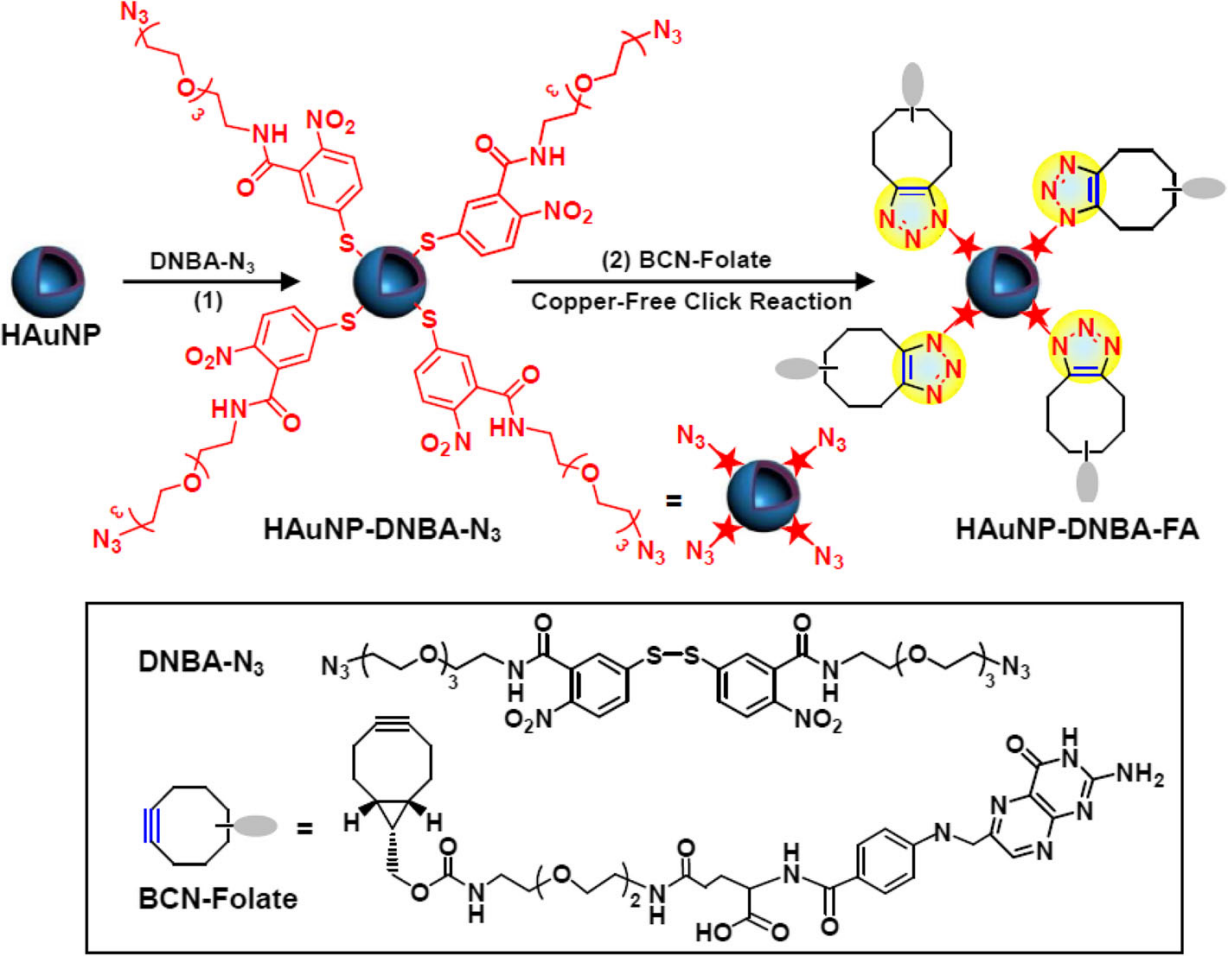
Modification of SERS nanoprobes with folate by copper-free click chemistry between azide and BCN for cancer cell imaging. Reproduced from reference [18] with permission

In 2017, Liu et al. developed folate receptor (FR)-targeted surface-enhanced Raman scattering (SERS) nanoprobes based on click chemistry [18] (Fig. 1). They synthesized hollow gold nanoparticles which can enhance Raman signals, and modified them with azide-labeled 5,5′-dithiobis(2-nitrobenzoic acid), a Raman active molecule by interaction between thiol and gold surface. Then, the azide groups on the surface of nanoparticles were conjugated to folate bicyclo[6.1.0]nonyne (BCN) derivatives, a kind of strain-promoted alkyne by click chemistry. The strongest Raman intensity peak at 1332 cm^− 1^ from the resulting nanoprobe was not changed after long term storage for three months. Both dark-field images and SERS images showed intense signal in folate receptor-overexpressed KB cells in different with the control Hela and A549 cells. The signals were not observed in case of using azide-modified nanoparticles without folate or folate-saturated KB cells showing that click chemistry enabled easy conjugation of folate to the nanoprobe without perturbation of its Raman imaging property.

In the same year, Chen et al. applied click chemistry to enhance targeting efficiency of nanogels to overcome multi-drug resistance (MDR). The synthesized biodegradable nanogel was composed of hydroxyethyl methacrylamide-oligoglycolates-derivatized poly(hydroxyethyl methacrylamide-co-N-(2-azidoethyl)methacrylamide) (p(HEMAm-co-AzEMAm)-Gly-HEMAm), and doxorubicin (DOX) was loaded in it [19]. After gelation, the surface of nanogel containing azide groups was decorated with folic acid (FA)-polyethylene glycol (PEG)-BCN via copper-free click chemistry. The nanogel backbone has pH sensitive hydrazine linkage between methacrylamide polymer and DOX, which can lead fast release of DOX in cancer cells. The nanogel showed faster cellular uptake in FA receptor-positive B16F10 cell line than FA receptor-negative A549 cell line due to the receptor-mediated endocytosis demonstrating successful conjugation of FA-PEG to nanogels by click chemistry. DOX was rapidly released in cytosol with the low pH environment and accumulated in nuclei. Furthermore, FA-modified nanogel showed lower resistance index than other non-targeted formulations and efficient killing of DOX-resistant 4 T1 cell by bypassing the drug efflux pumps of MDR.

Recently, Wei et al. synthesized nanoparticles composed of multiblock poly-urethanes (MPUs) which can switch tumor targeting upon pH and trigger drug release for tumor therapy and MR imaging [20]. The soft segments are composed of poly(ε-caprolactone) (PCL) and detachable poly(ethylene glycol)(PEG) with pH-sensitive benzoic-imine linkage which is cleavable under acidic condition like solid tumor microenvironment. The hard segments contain L-lysine ethyl ester diisocyanate (LDI) and cysteine-derived chain extender (Cys-PA) with clickable alkyn moieties. Copper-catalyzed click chemistry between azide and alkyne was used to conjugate the targeting ligands, folate groups to the nanoparticles after nanoparticle preparation. Cys-PA also has cleavable disulfide bonds which can trigger the core degradation and finally release drug within tumor cells after cellular uptake. Also, combination with super-paramagnetic iron oxide nanoparticles as contrast agents and doxorubicin, anticancer drug, within micelle core enable to exhibit MRI (magnetic resonance imaging) and cancer therapy. In vitro result shows excellent MRI contrast enhancement and the detachment of outer PEG in acidic condition, which can expose the clicked folate ligands for the enhanced cellular uptake. After that, elevated glutathione concentration in cytosol triggered reducible core degradation and enhanced drug release rate. Furthermore, MPUs showed efficient tumor-targeting in vivo during mice experiment resulting in successful anticancer therapy and MR imaging. In this paper, click chemistry also enabled easy post-conjugation of folate to MPUs similar with other papers.

**Fig. 2:**
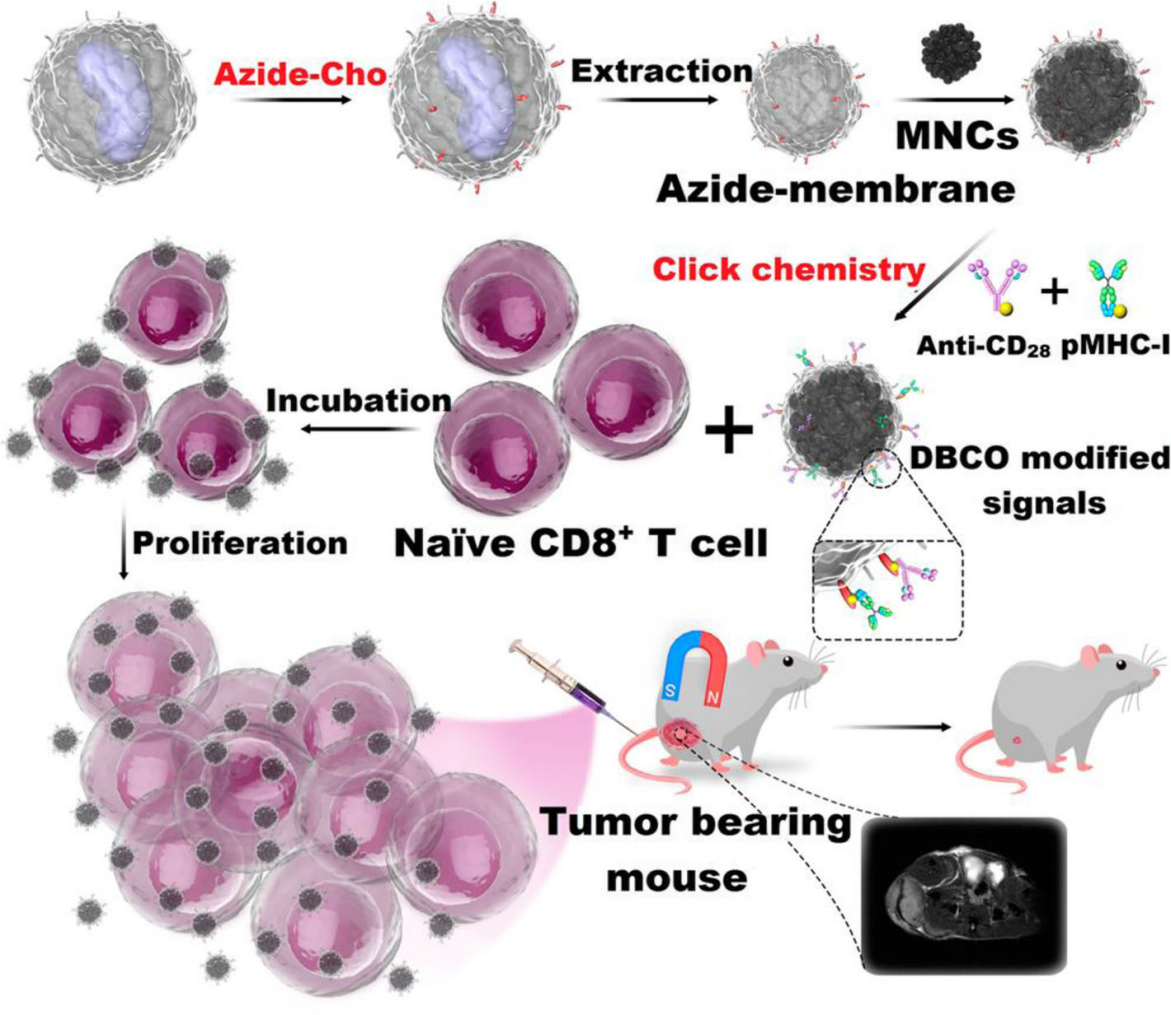
Decoration of T cell stimuli to leucocyte membranes by copper-free click chemistry between azide and DBCO for immunotherapy with adoptive T-cells. Reproduced from reference [21] with permission

In 2017, Zhang et al. modified their biomimetic magnetosomes based on artificial antigen-presenting cells (aAPCs) with stimulatory signals using copper-free click chemistry [21] (Fig. 2). With azide-choline, azide groups were introduced on the surface of leucocyte membranes via intrinsic biosynthesis. After extracting the inner materials of leucocyte, the pre-engineered aAPCs were decorated with dibenzocyclooctyne (DBCO)-modified T-cell stimulatory signals using click chemistry with azide groups. After these steps, T-cell stimulatory signals such as pMHC-I and anti-CD28 were localized on aAPCs to stimulate T cells. The major factor in T-cell-based therapy is ex vivo T-cell expansion, stimulation, and in vivo targeting performance. Synthesized aAPCs with T-cell stimuli was effective to antigen-specific cytotoxic T-cell (CTL) expansion in vitro and showed high affinity with CTLs, which could lead more CTLs at tumor site in vivo. Because aAPCs were also decorated with magnetic nanoparticle, aAPCs-CTL complexes could be monitored by MR imaging using T_2_ contrast signal intensity. Furthermore, aAPCs showed efficient adoptive T-cell-based anticancer immunotherapy in vivo during murine lymphoma model experiment. This result is a good example that click chemistry was also useful in immunotherapy with adoptive T-cells.

A recent study of Bao et al. showed another type of click chemistry reaction for nanoparticle synthesis [22]. Instead of using copper solution, they used laccase from Trametes versicolor, a copper-containing protein and hypothesized that laccase also can catalyze click reaction in water. They modified laccase with azide groups and synthesized amphiphilic polymers with terminal alkyne groups and thermo-sensitivity. The reaction between the two components resulted in self-assembled protein-polymer conjugate successfully by click chemistry. The enzyme activity was maintained during the reaction, so that it will be useful for enzyme immobilization. This report demonstrated that enzymatic click chemistry reaction is possible.

#### Application in targeted delivery of nanoparticle

In 2012, Koo et al. introduce an interesting tumor-targeting strategy for nanoparticles based on metabolic glycoengineering and click chemistry in vivo [23]. They artificially introduced the targetable azide group-containing glycans to tumor cell by the precursor, tetraacetylated N-azidoacetyl-d-mannosamine (Ac_4_ManNAz) based on metabolic glycoengineering. Then, DBCO-conjugated liposomes (DBCO-lipo) were treated to the tumor cells. DBCO groups strongly and specifically bound to the azide groups on the target cancer cell surface by copper-free click chemistry. In vitro study showed that the cellular uptake of DBCO-lipo was higher than the control PEG-lipo. In vivo study showed that the expression of azide groups on tumor tissue could be controlled by metabolic glycoengineeringin a dose-dependent manner. The enhanced tumor-targeting of DBCO-lipo was observed after intratumoral injection of Ac_4_ManNAz and intravenous injection of DBCO-lipo. This paper demonstrated that the distribution and tumor-targeting of nanoparticles could be controlled by click chemistry in vivo.

**Fig. 3:**
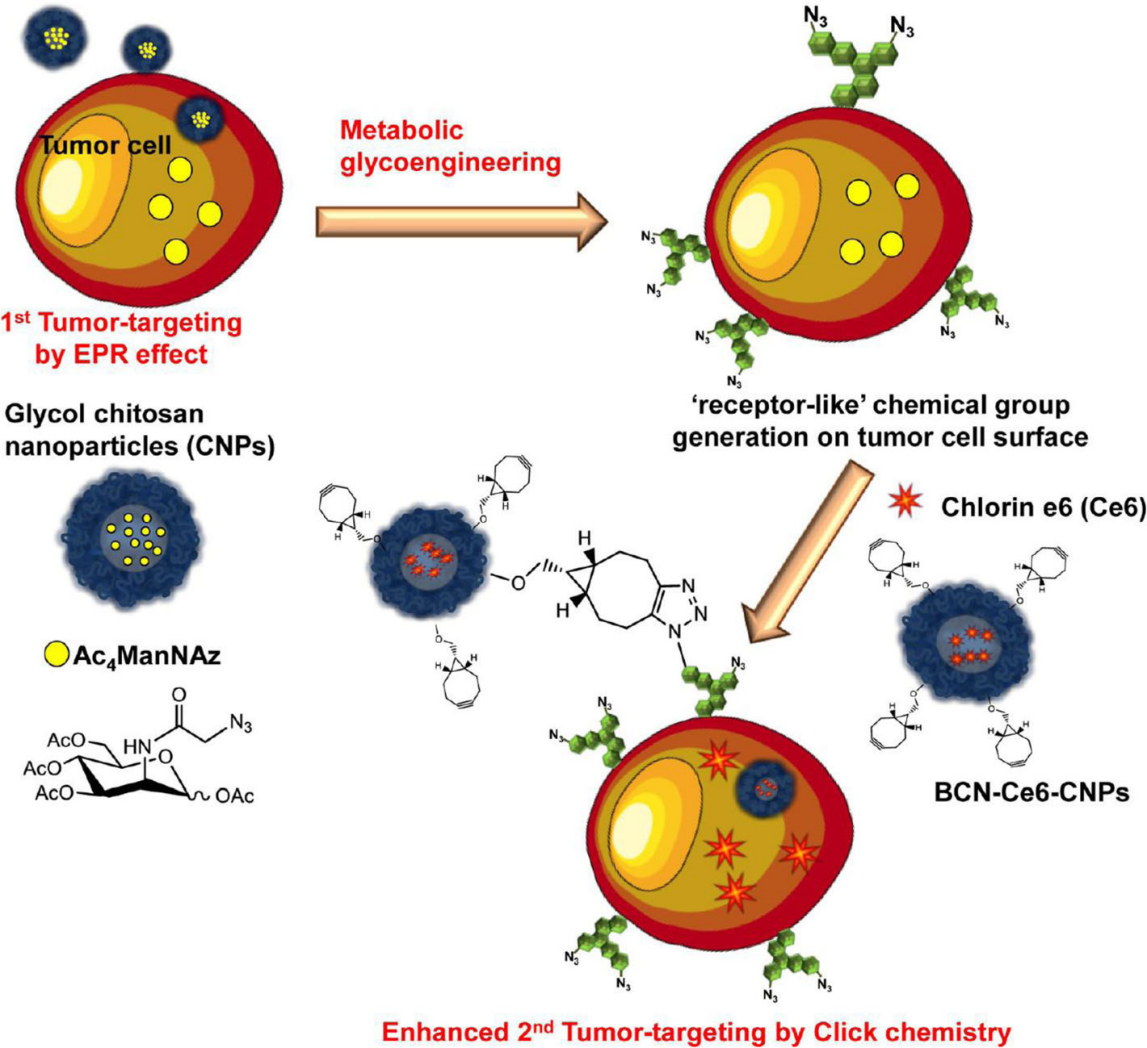
Two-step tumor-targeting of glycol chitosan nanoparticles via copper-free click chemistry in vivo between azide and BCN for photodynamic therapy. Reproduced from reference [24] with permission

In other paper, the same group made an effort to apply these results for drug delivery and tumor therapy in vivo [24] (Fig. 3). They used azide groups and bicyclo[6.1.0]nonyne (BCN) to enhance tumor-targeting by metabolic glycoengineering and click chemistry. They proposed two-step strategy. First, Ac_4_ManNAz, the azide precursor was loaded into glycol chitosan nanoparticles (CNP). After injection to tumor-bearing mice, the resulting Ac_4_ManNAz–CNPs were delivered to the tumor by EPR effect, afterward azide groups generated on the tumor cells by metabolic glycoengineering. Second, BCN-modified and chlorin e6 (Ce6)-loaded CNPs were injected intravenously to the same mice. After that, BCN groups on the surface of the BCN-Ce6-CNPs were conjugated to azide groups generated on the surface of the tumor by click chemistry in vivo. This click reaction could increase the accumulation of BCN-Ce6-CNPs in tumor tissue and resulted in efficient drug delivery and tumor therapy.

Recently, Wang et al. used peptide to generate the azide groups on the cancer cell surface specifically and induce more controlled click chemistry in vivo [25]. They used similar precursor, Ac_4_ManAz for metabolic glycoengineering, but they modified it with peptide sequence cleavable by histone deacetylase and cathepsin L (HDAC/CTSL). While the peptide is linked to the precursor, it cannot work. However, these enzymes are abundant in tumor tissue, so that the modified precursor could generate azide groups in tumor tissue after intravenous injection to the tumor-bearing mice. They also synthesized DBCO-DOX conjugate which can bind to the azide groups resulting in the enhanced accumulation of DOX in tumor tissue. The enhanced tumor targeting by click chemistry in vivo between azide and DBCO was proved in three kinds of tumor models including primary LS174T colon tumor, MDA-MB-231-triple-negative tumor, and 4 T1 lung metastases.

**Fig. 4:**
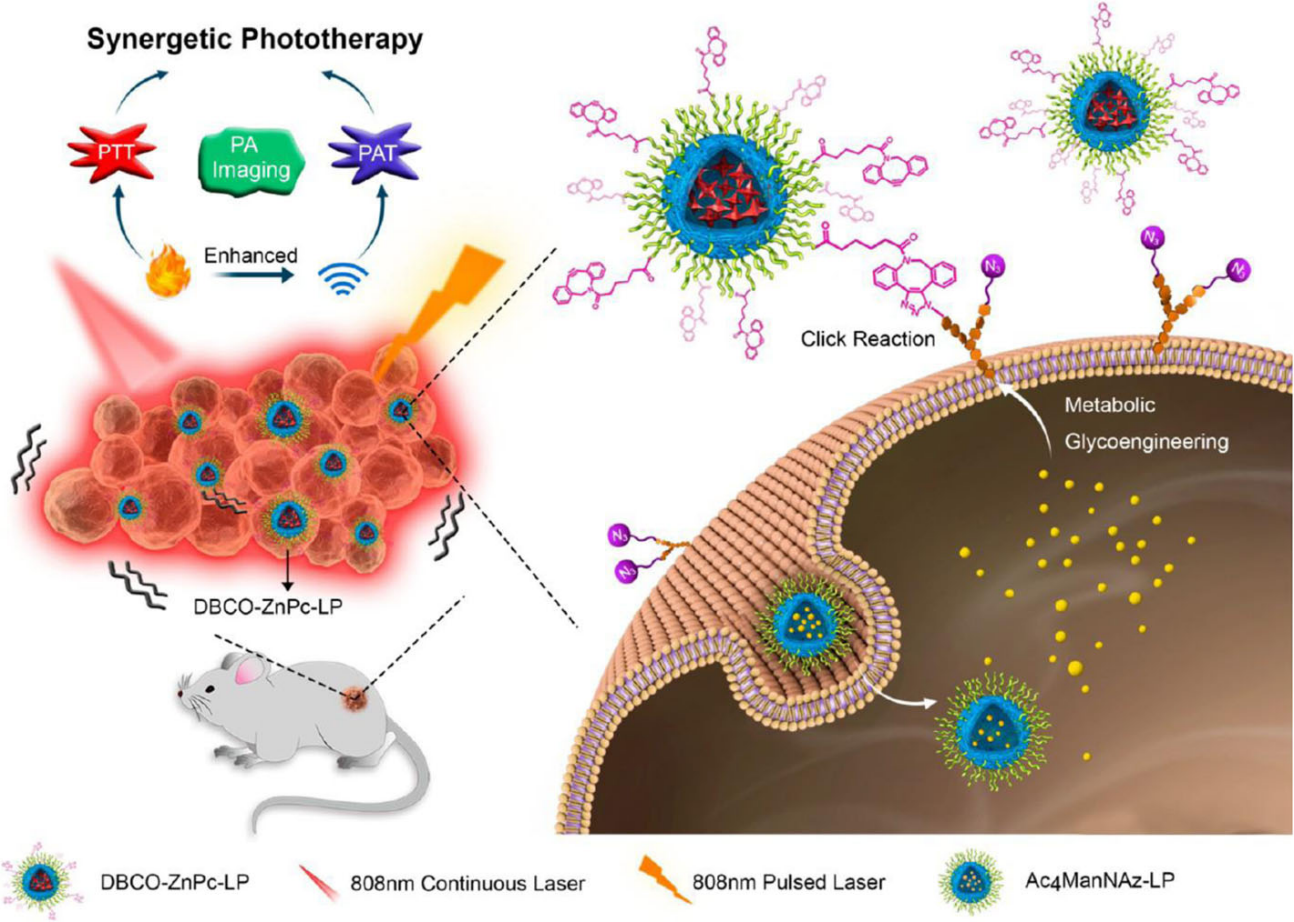
Two-step tumor-targeting of zinc (II)-phthalocyanine-loaded liposomes via copper-free click chemistry in vivo between azide and DBCO for photothermal therapy. Reproduced from reference [26] with permission

In 2017, Du L et al. applied click chemistry in vivo to deliver their multifunctional nanoplatforms for photothermal therapy and photoacoustic therapy (PAT) [26] (Fig. 4). They developed DBCO-modified zinc (II)-phthalocyanine-loaded liposome (DBCO-ZnPc-LP) and used two-step tumor-targeting strategy via metabolic glycoengineering and click chemistry. First, Ac_4_ManNAz-loaded liposome was intravenously injected to nude mouse, which can express azide group for chemical receptor on cell surface by metabolic glycoengineering as performed in the above paper by Koo et al. Then, after intravenous injection, the accumulation of DBCO-ZnPc-LP in tumor tissue was similarly increased by copper-free click chemistry in vivo between azide groups on cell surface and DBCO. The accumulated DBCO-ZnPc-LP in tumor tissue could generate large amount of heat and acoustic effect upon laser irradiation, which lead efficient PTT and PAT and destroyed tumor tissue. We expected that these kinds of tumor-targeting based on metabolic glycoengineering and click chemistry in vivo also can be applied to the other nanoparticles with functional groups for click chemistry on their surface [27].

## Conclusions

In summary, we introduced recent application of click chemistry in nanoparticle research. During modification of biological ligands on the surface of nanoparticle, the intrinsic property and function of both ligands and nanoparticles need to be preserved. Click chemistry is suitable for these purposes and used to attach ligands to nanoparticles easily. Furthermore, traditional chemical reactions were hard to occur in vivo because they use organic solvents, high temperature/pressure, or toxic catalysts. Click chemistry is free from these weak points, and it was successfully increased the binding between nanoparticles and target cells and enhanced their targeting after introduction to living body. In our opinion, current click reactions provide favorable specificity and reaction rate in aqueous condition, so that it can be applied easily in many studies in test tubes during synthetic steps. However, some complicated conditions including cell cytosol or in vivo need more careful approaches, because there are many factors to be considered. For example, nonspecific interactions with various kinds of natural proteins or secretion from body may be the problems to overcome. Particularly, many chemists are still developing more advanced click chemistry than before [28]. These trials will provide further increased specificity or reaction rate of click reactions and help solving the problems. Considering that, we expect that the potential and utility of click chemistry in biomedical science will be even greater in future.
